# Exploring the Link Between Special Educational Needs and Mental Health of Schoolchildren and Their Parents

**DOI:** 10.3390/children12030314

**Published:** 2025-02-28

**Authors:** Juan Manuel Núñez, Marián Pérez-Marín, Ana Soto-Rubio

**Affiliations:** 1 Faculty of Psychology and Speech Therapy, Universitat de València, 46010 Valencia, Spain; nujuanma@alumni.uv.es; 2 Department of Personality, Assessment and Psychological Treatments, Universitat de València, 46010 Valencia, Spain; marian.perez@uv.es; 3 Department of Developmental and Education Psychology, Universitat de València, 46010 Valencia, Spain

**Keywords:** child psychopathology, executive functions, infant psychopathology, parental mental health, special educational needs

## Abstract

Background/Objectives: The relationship between executive functions, special educational needs (SEN), and psychopathology in school-aged children is critical to the design of effective educational and therapeutic interventions. This study examines the connection between executive functions, SEN, schoolchildren’s psychopathology, and parental mental health. The objectives were to describe the psychopathological profiles of schoolchildren, to analyze the psychopathological differences between students with and without SEN, and to know the mental health status of parents of children with SEN. Methods: A total of 123 schoolchildren with and without SEN, together with their parents, participated in the study. Validated instruments were used to assess executive functions and child psychopathology, and an ad hoc register was used to assess parental mental health. In addition, sociodemographic and clinical data were collected. Results: The results revealed that children with SEN showed a significantly more affected psychopathological profile compared to their peers without SEN in all areas assessed. In addition, parents of children with SEN reported higher levels of emotional overburden (r = 0.39, *p* < 0.01). Deficits in executive functions, such as inhibition (r = 0.41, *p* < 0.01), working memory (r = 0.37, *p* < 0.01), and cognitive flexibility (r = 0.33, *p* < 0.05), were also found to be related to greater psychopathological problems in children. Conclusions: These findings underscore the importance of considering both executive functions and psychopathological profiles in designing educational and therapeutic interventions for children with SEN. It is recommended that intervention programs should comprehensively address the educational and emotional needs of children, as well as the well-being of their parents, with a specific focus on improving executive functions and reducing psychopathological disorders.

## 1. Introduction

Child mental health is a topic of growing global interest and concern due to its profound impact on children’s emotional, social, and academic development. According to UNICEF [1], one in seven children experiences a mental health disorder, highlighting an alarming reality. This figure underscores the urgent need for action from various societal actors, including families, educational institutions, and healthcare systems. Among this population, suicide ranks as one of the leading causes of death, emphasizing the necessity of comprehensive approaches to address mental health challenges in childhood. These issues not only affect children’s lives but also place significant strain on their families, disrupting daily dynamics and creating emotional, social, and economic challenges. Furthermore, the impact of these disorders on academic performance and the ability to form social relationships compounds the challenge, jeopardizing children’s quality of life and holistic development [1].

Child psychopathology is traditionally categorized into two major groups: externalizing and internalizing symptoms [2]. Externalizing symptoms, such as hyperactivity, aggression, and conduct disorders, are often more visible and disruptive. Conditions like Attention-Deficit/Hyperactivity Disorder (ADHD) and conduct disorders exemplify these symptoms, directly affecting academic performance and complicating social interactions [3]. These symptoms have significant repercussions in educational settings, not only for the affected child but also for peers and teachers, who must adapt pedagogical strategies to meet these needs. In contrast, internalizing disorders, such as anxiety, depression, and phobias, tend to be less apparent and harder to detect. These conditions hinder children’s participation in daily activities, complicate peer relationships, and impede academic performance, with potentially long-term consequences if left unaddressed [4]. The complexity of these disorders highlights the importance of training health and education professionals to effectively identify and manage these challenges. Notably, both categories of disorders, despite their differing manifestations, require early and appropriate intervention to prevent adverse outcomes in adulthood [5].

In this context, executive functions (EFs) play a crucial role in emotional regulation and impulse control, serving as key mechanisms for managing both externalizing and internalizing disorders [6]. EFs are higher-order cognitive skills, including working memory, inhibition, and cognitive flexibility, among others, which enable individuals to manage appropriate behaviors in complex and novel situations [7,8]. These skills are essential for developing academic and social competencies, particularly in an increasingly demanding cognitive and emotional environment. Deficits in these functions are commonly observed in children with conditions like ADHD or mental health issues such as anxiety and depression. These deficits hinder emotional regulation, impulse control, and adaptability to new situations, exacerbating symptoms of childhood disorders and interfering with learning and social interactions [9].

The growing prevalence of students with special educational needs (SEN) further highlights the importance of addressing these challenges. SEN encompasses a wide range of conditions, including physical, sensory, cognitive, mental, emotional, social, and learning difficulties. According to Spain’s Organic Law for the Improvement of Educational Quality (LOMCE) [10], students with SEN are those whose personal circumstances create barriers to their holistic development, both academically and socially. During the 2022–2023 academic year, 12% of students were identified as having SEN, with 27.2% having disabilities or severe disorders and 72.8% representing other specific educational support needs [11]. Over the past decade, the school population with SEN has increased by 42.1% [12]. This rise may be attributed to factors such as greater social awareness, advances in identifying and diagnosing conditions affecting learning, and broader recognition of temporary or permanent needs hindering childhood development.

The influence of SEN also imposes considerable psychological stress on parents, particularly mothers, who often assume primary caregiving responsibilities. This stress arises not only from the emotional demands of raising a child with disabilities but also from concerns about their child’s future and the daily challenges of schooling and social adaptation. Recent studies [13] indicate that mothers of children with SEN experience high levels of stress, influenced by their perceptions of their child’s characteristics and the availability of family support. This support, encompassing financial resources and social networks, can make a crucial difference in parental experiences. Evidence suggests that parental stress increases significantly when parents perceive insufficient resources to meet their child’s educational needs [14]. This can lead to a decline in parents’ emotional well-being, as they feel overwhelmed by daily caregiving demands, affecting their overall mental health and quality of life [15].

Interventions designed for children with SEN must address not only the children’s needs but also those of their parents, providing emotional support and strategies to reduce stress and enhance family well-being [15]. In this regard, interventions focused on strengthening EFs are particularly relevant, as these skills are fundamental for children to adapt to school and social environments, solve problems, and regulate emotions [16]. Evidence suggests that these strategies not only benefit the directly affected students but also foster a culture of inclusion that positively impacts the entire educational community [17].

This study aimed to examine the relationship between EFs, SEN, student psychopathology, and parental mental health, based on the hypothesis that SEN has a distinctive impact on both students and their parents. Specifically, the objectives were to describe the psychopathological profiles of students, analyze differences in psychopathology between students with and without SEN, and evaluate the mental health status of parents of children with SEN.

## 2. Materials and Methods

### 2.1. Sample

The study sample consisted of 123 primary school students from Castellón, Spain, aged 6 to 12 years (mean = 8.61; SD = 1.8). In terms of sex, 53.6% were boys. According to the information provided by the families, 3.2% of the students had a diagnosed medical condition (such as asthma, diabetes, or epilepsy) and 11.2% had a psychological condition (such as anxiety, neurodevelopmental disorders, or mood disorders). In addition, according to teacher reports, 31% (n = 38) of the students were identified as having SEN.

Data collection involved direct input from the students, complemented by additional information from their teachers and parents. The inclusion criteria were as follows:

(a) Enrollment in elementary education.
(b) For children with SEN, possessing the cognitive abilities required to complete the assessment was mandatory.
(c) Voluntary participation.

The schools’ reports on adaptive measures for children with SEN served as the basis for diagnosing SEN.

This study received approval from the ethics committee of the University of Valencia (reference UV-INV_ETICA-3119648), and the necessary permissions to assess the schoolchildren were obtained from the Conselleria de Educación de la Comunidad Valenciana. The guidelines established by the Personal Data Protection Act (LOPD) 3/2018 of December 5 were strictly adhered to.

### 2.2. Instruments

The Behavior Rating Inventory of Executive Function (BRIEF-2) [18], the Spanish version validated by Gioia et al. [19]: This tool assesses executive functions in children aged 5 to 18 years based on reports from school and family contexts. Each report includes 63 items with three response options (never, sometimes, and often). The instrument evaluates the following components: inhibition (impulse control and the ability to suppress automatic or inappropriate responses), self-control (the ability to self-evaluate and regulate behavior), cognitive flexibility (adaptability to changes and the ability to switch between tasks or thoughts), emotional control (effective management of emotional responses), initiative (the ability to independently start tasks or activities), working memory (retention and temporary manipulation of information for complex tasks), planning and organization (creating structured strategies to complete tasks efficiently), task monitoring (maintaining attention and tracking progress on a task), and organization of materials (order and management of personal belongings and materials). Additionally, it provides composite indices: the behavioral regulation index (impulse control and behavior regulation), the emotional regulation index (the ability to manage emotions), the cognitive regulation index (management of cognitive processes), and the Global Executive Composite (a global measure of executive function performance).

Screening for Emotional and Behavioral Problems in Children (SPECI) validated for the Spanish population [20]: This questionnaire is designed to quickly and effectively identify emotional and behavioral difficulties in children aged 5 to 12, covering students from the 3rd grade of Infant Education to the 6th grade of Primary Education. Completed by teachers, it assesses the frequency of observable behaviors in the school setting through a concise and structured format. The SPECI includes 10 items that describe behaviors indicative of various challenges and provides three overarching scales: internalizing problems, reflecting emotional challenges, such as anxiety and social withdrawal; externalizing problems, encompassing disruptive or aggressive behaviors; and a total score, summarizing the overall severity of the identified issues.

Developmental Profile 3 (DP-3) [21], Spanish validated version [22]: The DP-3 is a brief assessment tool that measures five key areas of child development: cognitive, motor, socioemotional, communication, and adaptive behavior, through 180 items. These areas help identify potential delays or difficulties in a child’s overall development. It is designed for children from birth to 12 years and 11 months and is useful in both clinical and educational contexts. The DP-3 offers two application methods: a parent interview or a self-administered questionnaire, the latter being the option used in the present study.

Ad hoc registers: Ad hoc registers were drawn up to obtain the sociodemographic and clinical data on the children. Specifically, the variables collected were age and sex, academic year, medical and psychological diagnosis, and presence/absence of SEN (according to the school report on adaptation measures for children with SEN). At the same time, parents’ perceived mental health was assessed using an ad hoc 5-point Likert-type scale, where 1 represented “very bad” and 5 “very good”. Participants rated their status in the following areas: anti-anxiety, depression, general well-being, physical overburden, emotional overburden, and total overburden. This measure identified stress levels and well-being related to their experience as caregivers.

## 3. Procedure

The procedure employed in this study was carefully designed to ensure the validity and reliability of the data, minimizing potential sources of error. To this end, four public and private schools in Castellón (Spain) were randomly selected, and an invitation to participate in the research was sent to them via email. Subsequently, personal interviews were conducted with those who expressed interest, providing detailed information about the study’s objectives and participation conditions.

After obtaining institutional approval, a training session was organized for teaching staff and families. This session emphasized the voluntary nature and anonymity of responses, ensuring a clear understanding of the procedure. During this phase, families were given detailed instructions for completing the questionnaires, which were also included within the instruments themselves. Those who decided to participate signed informed consent forms and returned the completed questionnaires to the school, where they were attached to each student’s individual file along with the assessment battery completed by the teacher.

Data collection was conducted in a single session during the students’ homeroom period, with the tutor supervising the process without interfering with the students’ responses. In total, information was obtained from 123 students, collected through their teachers, parents, and the students themselves. Additionally, data on the mental health of 88 parents were collected through ad hoc registers. To ensure the independence of sources and avoid bias, the data provided by families and teachers remained completely anonymous and were not cross-referenced.

The diagnosis of SEN was carried out by the school counselor, an authorized professional within the Spanish educational system for conducting such evaluations. Regarding the assessment tools, widely validated and well-established instruments for Spanish-speaking populations were chosen. The Prediscal was selected for its high ecological validity and efficiency in detecting learning difficulties. Additionally, the SPECI and BRIEF-2 questionnaires, in their latest Spanish versions, were used due to their strong psychometric properties and extensive application in educational settings.

To ensure uniformity in the administration and interpretation of the instruments, all evaluators received prior training and participated in regular supervision meetings. This structured and collaborative approach contributed to minimizing errors and enhancing the reliability of the data obtained, ensuring the study’s overall quality.

### Statistical Analysis

The study of mean differences was carried out using a categorical independent variable, in which the criterion variable was the presence or absence of SEN, categorized into two groups: with SEN and without SEN. Differences between these groups were evaluated using the Student’s *t*-test for independent samples. Levene’s test was applied for homogeneity of variances, and the effect size was calculated using Cohen’s d in the cases in which the means of the independent samples were compared.

The dependent variables analyzed in the study were executive functions, students’ psychopathology, and the parents’ mental health. Pearson’s correlation coefficient was also used to explore possible relationships between these dependent variables and other variables in the study. All statistical analyses were performed using SPSS version 29.0 software.

## 4. Results

### 4.1. Sample Description

The study included 123 schoolchildren aged 6 to 12 years (M = 8.61; SD = 1.8), of whom 53.6% were male. Among these, 3.2% had a medical diagnosis and 11.2% had a psychological diagnosis. According to teachers’ reports, 31% of students were identified as having SEN. Regarding specialized support within the school, 7.2% received therapeutic pedagogy and 8% accessed auditory and speech therapy. Outside the school environment, the majority of children (60%) did not receive any specialized care, while only 3.2% received psychological services (see Table 1).

### 4.2. Parents’ Demographics

A total of 88 parents participated in the study, most of whom were mothers (82.9%). Their ages ranged from 29 to 57 years (M = 41; SD = 5.25). Most participants were married (48%) and had an educational level equivalent to vocational training or high school (33.2%) or university studies (24.8%). In terms of employment, a significant proportion were actively working, either under a permanent contract (28.8%) or as self-employed workers (16.8%) (see Table 2 for details).

### 4.3. Correlations Between Variables

As shown in Table 3, a positive correlation was identified between children’s externalizing behavior problems and parents’ emotional overburden (r = 0.304, *p* ≤ 0.05). No significant relationships were observed between internalizing behavior problems or the global psychopathology index and caregivers’ mental health.

Regarding socioemotional development, a significant positive relationship was found between the global psychopathology index and children’s socioemotional development (r = 0.319, *p* ≤ 0.05).

Regarding executive functions, working memory was the only function that showed a significant association with psychopathology in both family and school contexts. In the school context, working memory was positively associated with all three dimensions evaluated: internalizing problems (r = 0.599, *p* ≤ 0.01), externalizing problems (r = 0.534, *p* ≤ 0.01), and the global psychopathology index (r = 0.785, *p* ≤ 0.01). In the family context, this relationship was observed only with internalizing problems (r = 0.343, *p* ≤ 0.05) and the global psychopathology index (r = 0.310, *p* ≤ 0.05).

In teacher evaluations, other executive functions, such as cognitive flexibility (r = 0.695, *p* ≤ 0.05), initiative (r = 0.706, *p* ≤ 0.05), planning and organization (r = 0.815, *p* ≤ 0.01), and task supervision (r = 0.814, *p* ≤ 0.01), showed positive correlations with all three dimensions of psychopathology. Conversely, skills such as inhibition (r = 0.895, *p* ≤ 0.05), behavioral regulation (r = 0.697, *p* ≤ 0.01), and self-monitoring (r = 0.826, *p* ≤ 0.01) showed significant positive relationships only with externalizing problems and the global psychopathology index, with no associations observed with internalizing problems.

### 4.4. Correlation Analysis by SEN Status

To better understand these associations, a detailed correlation analysis was conducted for schoolchildren with and without SEN.

### 4.5. Children with SEN

Significant positive correlations were observed exclusively in the school context between executive functions and dimensions of psychopathology. Internalizing psychopathology was positively correlated with initiative (r = 0.706, *p* ≤ 0.05), working memory (r = 0.662, *p* ≤ 0.05), planning and organization (r = 0.737, *p* ≤ 0.01), task supervision (r = 0.689, *p* ≤ 0.05), the cognitive regulation index (r = 0.687, *p* ≤ 0.05), and the global executive function index (r = 0.659, *p* ≤ 0.05). Similarly, internalizing psychopathology showed positive correlations with parental physical overburden (r = 0.775, *p* ≤ 0.05) and emotional overburden (r = 0.808, *p* ≤ 0.01).

Externalizing psychopathology was positively correlated with planning and organization (r = 0.707, *p* ≤ 0.05) and inversely related to motor development (r = −0.865, *p* ≤ 0.05). Regarding the global psychopathology index, positive correlations were identified with inhibition (r = 0.895, *p* ≤ 0.05), working memory (r = 0.880, *p* ≤ 0.05), planning and organization (r = 0.815, *p* ≤ 0.01), task supervision (r = 0.814, *p* ≤ 0.01), the cognitive regulation index (r = 0.887, *p* ≤ 0.01), and the global executive function index (r = 0.849, *p* ≤ 0.01). Negative correlations were found with motor development (r = −0.758, *p* ≤ 0.05) and positive correlations with parental physical overburden (r = 0.728, *p* ≤ 0.05) and emotional overburden (r = 0.671, *p* ≤ 0.05).

Planning and organization emerged as the only executive functions correlated with all three dimensions of psychopathology (see Table 4).

### 4.6. Children Without SEN

In children without SEN, positive correlations were observed between executive functions and dimensions of psychopathology in both school and family contexts, with working memory being the only executive function showing correlations in both contexts (see Table 5).

### 4.7. Group Comparisons: Children with and Without SEN

To examine differences between groups, comparisons were made between children with and without SEN (Table 6). Significant differences were found in internalizing psychopathology (t = −3.184, *p* = 0.044, d = 1.044), externalizing psychopathology (t = −1.946, *p* = 0.038, d = 1.275), and total psychopathology (t = −3.620, *p* = 0.022, d = 1.041), with children with SEN scoring higher in all domains.

### 4.8. Influence of Gender on Mental Health and Psychopathology

To explore the potential influence of gender, analyses were conducted based on the gender of the children and the parents.

### 4.9. Children’s Gender

No significant differences were observed in most variables evaluated. However, regarding parental mental health, a significant difference was found in overburden based on the child’s gender: parents of male children reported a greater perception of overburden (t = 2.212, *p* = 0.018, d = 2.509), indicating a more significant impact on this group (see Table 7).

### 4.10. Parents’ Gender

When analyzing differences based on the gender of the parent, fathers reported higher depression scores compared to mothers (t = −2.114, *p* = 0.017, d = 1.153), reflecting poorer self-reported emotional well-being among fathers (see Table 8).

Finally, no significant differences were found in children’s psychopathology based on gender.

## 5. Discussion

This study aimed to analyze the relationship between executive functions (EFs), SEN, students’ psychopathology, and parental mental health. The findings reveal critical insights that align with and extend the existing literature, emphasizing the multifaceted challenges faced by children with SEN and their families.

The results highlight a significant prevalence of psychopathological disorders among students with SEN, particularly internalizing problems. This finding corroborates prior research indicating that children with SEN are at heightened risk for emotional difficulties, such as anxiety and depression, which may be less visible but equally debilitating compared to externalizing problems (e.g., hyperactivity or aggression) [23,24]. Unlike studies that predominantly focus on externalizing issues [25], our findings underscore the need for early identification and intervention targeting emotional vulnerabilities in children with SEN. The methodological emphasis on validated tools like the BRIEF-2 and SPECI may account for the nuanced detection of internalizing symptoms in this study.

The observed differences in psychopathology between students with and without SEN are consistent with the literature documenting the broader cognitive, emotional, and social challenges faced by children with SEN [26]. Notably, children with SEN scored significantly higher on all dimensions of psychopathology, underscoring the compounded difficulties they encounter. While this aligns with previous findings, it is essential to acknowledge potential mediating factors, such as the availability of school or family support, which were not extensively explored in this study. Future research could delve deeper into how these external influences modulate the relationship between SEN and psychopathology.

The study’s findings also shed light on the emotional burden experienced by parents of children with SEN, particularly mothers. Higher levels of emotional and physical overburden were reported, aligning with evidence that caregiving demands significantly impact parental mental health. This underscores the necessity of designing interventions that support not only children but also their caregivers. The inclusion of structured support networks and resources, such as counseling or peer support groups, could mitigate the stress experienced by parents and improve overall family dynamics. Recent studies suggest that access to socioeconomic resources and institutional support can play a crucial role in alleviating caregiver burden [27], an area warranting further exploration.

The relationship between deficits in executive functions and increased psychopathology in children with SEN is a key finding of this study. Specifically, impairments in working memory, inhibition, and cognitive flexibility were significantly associated with higher levels of psychopathology. These results align with research linking EF deficits to emotional dysregulation and behavioral challenges in children with neurodevelopmental conditions [28]. Moreover, planning and organization emerged as pivotal executive functions correlated with all dimensions of psychopathology, highlighting their central role in adaptive functioning. These findings reinforce the importance of incorporating EF training into intervention programs for children with SEN.

The study’s findings have several practical implications. Schools should implement targeted programs to enhance executive functions, focusing on skills such as working memory, cognitive flexibility, and planning. These interventions can be integrated into classroom activities or delivered through specialized support services. Educators should also be equipped with tools and strategies to identify and manage emotional and behavioral challenges in students with SEN. Training programs could emphasize recognizing early signs of internalizing and externalizing psychopathology and adapting teaching methods accordingly. Additionally, developing resources for parents, such as stress management workshops or access to mental health services, is crucial. Empowering parents with coping strategies can alleviate their emotional burden and enhance their capacity to support their children effectively. Greater coordination between educational and mental health services is also essential to provide comprehensive support. Multi-disciplinary teams comprising educators, psychologists, and social workers could facilitate holistic interventions addressing both academic and emotional needs.

While the study provides valuable insights, several limitations should be acknowledged. The sample size, limited to schools in Castellón, Spain, may restrict the generalizability of the findings. Expanding the sample to include diverse geographic and cultural contexts would enhance the study’s applicability. Additionally, the cross-sectional nature of the study limits the ability to infer causality, and longitudinal studies are needed to examine the long-term impact of executive function deficits and psychopathology on children with SEN. The study did not account for socioeconomic variables, which could significantly influence the observed relationships, so future research should incorporate these factors to provide a more comprehensive understanding. Moreover, reliance on parent-reported measures of mental health may have introduced bias. Combining self-reports with objective assessments could yield more reliable data.

Future research should explore several areas. Evaluating the impact of executive function training programs on both academic and emotional outcomes for children with SEN is critical. Investigating how family structure, support networks, and socioeconomic resources influence parental mental health and child outcomes is also important. Examining how cultural factors shape the relationship between SEN, psychopathology, and executive functions could provide valuable insights. Furthermore, assessing the potential of digital tools and applications to support executive function development and parental well-being could open new avenues for intervention and support.

## 6. Conclusions

This study reinforces the importance of addressing emotional and behavioral difficulties in children with SEN within the school context. The findings highlight that these problems not only affect academic performance but also have significant implications for the child’s social and emotional adaptation and family dynamics.

The high prevalence of both internalizing and externalizing psychopathologies among students with SEN underscores the need for interventions that prioritize not only emotional well-being but also the development of executive functions, which play a crucial role in self-regulation and adaptive behavior, alongside academic performance. Furthermore, the high levels of emotional and physical overburden reported by parents highlight the importance of designing support strategies that include families as a key component of the intervention process.

From a practical perspective, this study’s results emphasize the urgency of providing teachers with tools and training to manage emotional and behavioral problems in the classroom. In addition, the findings suggest the importance of promoting awareness and training in identifying signs of psychopathology, which could enhance early detection and intervention efforts. They also call for greater coordination between the educational system and mental health services to ensure a comprehensive approach.

Future research should explore the longitudinal impact of emotional and behavioral problems on the development of children with SEN and the effectiveness of intervention programs focused on integrating emotional skills development, executive function training, and caregiver support.

This study underscores the intricate interplay between executive functions, psychopathology, and family dynamics in children with SEN. The findings highlight the urgent need for comprehensive, multi-faceted interventions that address the cognitive, emotional, and social needs of children and their families. By fostering collaboration between educational and mental health systems, stakeholders can create inclusive environments that promote the well-being and development of children with SEN and their caregivers. Future research should build on these findings to advance evidence-based practices and policies that support this vulnerable population.

## Figures and Tables

**Table 1:** Specialized care received outside the school context.

Type of Care	n	Percentage
Without attention	75	60
Psychology	4	3.2
Speech therapy	1	0.8
Pedagogy	3	2.4
Interdisciplinary	3	2.4

**Table 2:** Sociodemographic variables of parents.

Variable	N	Percentage
Civil status		
Single	6	4.8
Married	61	48.8
Divorced	10	8.0
Widower	2	1.6
Living as a couple	7	5.6
Employment situation		
Non-temporary contract	36	28.8
Temporary contract	7	5.6
Self-employed	21	16.8
Official	6	4.8
Unemployed	15	12.0
Other	1	0.8
Education level		
Without studies	3	2.4
Mandatory studies	9	7.2
Bachelor/professional cycles	42	33.6
University studies	31	24.8
Post-university studies	1	0.8

**Table 3:** Correlations between the variables studied.

		Internalizing Psychopathology	Externalizing Psychopathology	Total Psychopathology
	EFs in family context			
BRIEF-2	Working memory	0.343 *		0.310 *
	EFs in school context			
	Working memory	0.599 **	0.534 **	0.785 **
	Flexibility			
	Inhibition			
	Planning and organization	0.587 **	0.655 **	0.670 **
	Task supervision	0.507 **	0.547 **	0.651 **
	Self-supervision			
	Organization of materials	0.268 *	0.628 **	0.456 **
	GI. Behavioral regulation		0.697 **	0.399 **
	GI. Emotional regulation	0.366 **	0.292 *	0.492 **
	GI. Cognitive regulation	0.615 **	0.624 **	0.765 **
	GI. Executive functions		0.685 **	0.374 **
	Evolutionary development			
DP-3	Social development index			0.319 *
	Parental mental health			
Ad hoc	Emotional overburden parents		0.304 *	

Notes: * *p* ≤ 0.05, ** *p* ≤ 0.01; EFs = executive functions, GI = global index, BRIEF-2: Behavior Rating Inventory of Executive Function, DP-3: Developmental Profile 3.

**Table 4:** Correlations between the variables studied exclusively in schoolchildren with special educational needs.

		Internalizing Psychopathology	Externalizing Psychopathology	Total Psychopathology
	EFs in school context			
BRIEF-2	Initiative	0.706 *		
	Flexibility			0.695 *
	Inhibition			0.895 *
	Working memory	0.662 *		0.880 *
	Planning and organization	0.737 **	0.707	0.815 **
	Task supervision	0.689 *		0.814 **
	Cognitive regulation index	0.687 *		0.887 *
	GI. Executive functions	0.659 *		0.849 **
	Evolutionary development			
DP-3	Motor development		−0.865	−0.758
	Parental mental health			
Ad hoc	Physical overburden	0.775 *		0.728 *
	Emotional overburden	0.808 **		0.671 *

Notes: * *p* ≤ 0.05, ** *p* ≤ 0.01; EFs = executive functions, GI = global index, BRIEF-2: Behavior Rating Inventory of Executive Function, DP-3: Developmental Profile 3.

**Table 5:** Correlations between the variables studied exclusively in schoolchildren without special educational needs.

		Internalizing Psychopathology	Externalizing Psychopathology	Total Psychopathology
	EFs in family context			
BRIEF-2	Working memory	0.326 *		
	EFs in school context			
	Inhibition		0.884 **	
	Self-supervision		0.826 **	0.321 *
	Initiative	0.301		0.268 *
	Flexibility		0.329 *	0.358 **
	Emotional control		0.361 *	0.358 **
	Working memory		0.478 **	
	Planning and organization		0.555 **	
	Task supervision		0.411 **	
	Organization of materials		0.794 **	
	Behavioral regulation index		0.895 **	0.266 *
	Emotional regulation index		0.392 **	0.415 **
	Cognitive regulation index		0.611 **	0.313 *
	GI. Executive functions		0.813 **	0.377 **
	Evolutionary development			
DP-3	Social development			0.321 *

Notes: * *p* ≤ 0.05, ** *p* ≤ 0.01; EFs = executive functions, GI = global index, BRIEF-2: Behavior Rating Inventory of Executive Function, DP-3: Developmental Profile 3.

**Table 6:** Comparison of means between students with SEN and without SEN in schoolchildren’s psychopathology and parents’ mental health.

		Without SEN (n = 57) (Mean, SD)	With SEN (n = 12) (Mean, SD)	*t*	*p*	*d*
Ad hoc	Emotional overload	4.98; 2.804	6.53; 3.151	−2.054	0.022 *	2.883
SPECI	P. Internalizing	0.31; 0.730	2.17; 1.992	−3.184	0.044 *	1.044
	P. Externalizing	0.33; 0.852	1.75; 2.491	−1.946	0.038 *	1.275
	P. Total	0.58; 0.731	2.67; 1.969	−3.620	0.022 *	1.041

Notes: * *p* ≤ 0.05, P. = problem; Cohen’s d: small TE ≈ 0.20, moderate TE ≈ 0.50, large TE ≈ 0.80. SPECI: Screening for Emotional and Behavioral Problems in Children.

**Table 7:** Comparison of means for the mental health of parents and psychopathology of minors according to the gender of the schoolchild.

		Male (n = 42) (Mean, SD)	Female (n = 43) (Mean, SD)	*t*	*p*	*d*
Ad hoc	Overburden	5.64, 2.694	4.49, 2.313	2.212	0.018	2.509

Notes: Cohen’s d: small TE ≈ 0.20, moderate TE ≈ 0.50, large TE ≈ 0.80.

**Table 8:** Comparison of means for parental mental health and psychopathology of minors according to the gender of the parent.

		Mother (n = 72) (Mean, SD)	Father (n = 13) (Mean, SD)	*t*	*p*	*d*
Ad hoc	Depression	1.49, 1.113	2.23, 1.363	−2.114	0.017	1.153

Notes: Cohen’s d: small TE ≈ 0.20, moderate TE ≈ 0.50, large TE ≈ 0.80.

## Data Availability

The data presented in this study are available on request from the corresponding author. They are not publicly available due to data privacy and confidentiality.
